# Report of a Suit for Libel

**Published:** 1850-09

**Authors:** 


### ARTICLE VI.

*Report of the Trial. The People* vs. *Dr. Horatio N. Loomis, for Libel. Tried at the Erie County Oyer and Terminer, June* 24th, 1850. Buffalo: Jewett, Thomas & Co.; pp. 50; 8vo.

Our readers doubtless remember that a great noise was made in Buffalo last Spring, by divers fastidious medical gentlemen, whose modest sensibilities received a severe shock on the occasion of Prof. White's giving a clinical lecture on obstetrics. The gentlemen of the graduating class in the medical department of the University of Buffalo, were admitted to attend a case of labor, whereat many of the profession in the city professed themselves immensely scandalized. Dr. Loomis published a paragraph on the subject, in one of the city papers, for which he was indicted for libel, and this pamphlet contains a record of the trial, which resulted in a verdict of "not guilty." After reading the Judge's charge to the jury, we are free to confess that we esteem the verdict a singular one. Those of our readers who have curiosity enough to wade through the details of this trial, can probably obtain a copy on application to Prof. White, at Buffalo.

